# Characterization of the interactome profiling of *Mycoplasma fermentans* DnaK in cancer cells reveals interference with key cellular pathways

**DOI:** 10.3389/fmicb.2022.1022704

**Published:** 2022-10-28

**Authors:** Sabrina Curreli, Francesca Benedetti, Weirong Yuan, Arshi Munawwar, Fiorenza Cocchi, Robert C. Gallo, Nicholas E. Sherman, Davide Zella

**Affiliations:** ^1^Institute of Human Virology, University of Maryland School of Medicine, Baltimore, MD, United States; ^2^Department of Medicine, University of Maryland School of Medicine, Baltimore, MD, United States; ^3^Department of Biochemistry and Molecular Biology, University of Maryland School of Medicine, Baltimore, MD, United States; ^4^Biomolecular Analysis Facility Core, School of Medicine, University of Virginia, Charlottesville, VA, United States

**Keywords:** *Mycoplasma fermentans*, chaperone protein, DnaK protein, proteomics, cellular pathways

## Abstract

Chaperone proteins are redundant in nature and, to achieve their function, they bind a large repertoire of client proteins. DnaK is a bacterial chaperone protein that recognizes misfolded and aggregated proteins and drives their folding and intracellular trafficking. Some Mycoplasmas are associated with cancers, and we demonstrated that infection with a strain of *Mycoplasma fermentans* isolated in our lab promoted lymphoma in a mouse model. Its DnaK is expressed intracellularly in infected cells, it interacts with key proteins to hamper essential pathways related to DNA repair and p53 functions and uninfected cells can take-up extracellular DnaK. We profile here for the first time the eukaryotic proteins interacting with DnaK transiently expressed in five cancer cell lines. A total of 520 eukaryotic proteins were isolated by immunoprecipitation and identified by Liquid Chromatography Mass Spectrometry (LC-MS) analysis. Among the cellular DnaK-binding partners, 49 were shared between the five analyzed cell lines, corroborating the specificity of the interaction of DnaK with these proteins. Enrichment analysis revealed multiple RNA biological processes, DNA repair, chromatin remodeling, DNA conformational changes, protein-DNA complex subunit organization, telomere organization and cell cycle as the most significant ontology terms. This is the first study to show that a bacterial chaperone protein interacts with key eukaryotic components thus suggesting DnaK could become a perturbing hub for the functions of important cellular pathways. Given the close interactions between bacteria and host cells in the local microenvironment, these results provide a foundation for future mechanistic studies on how bacteria interfere with essential cellular processes.

## Introduction

DnaK is a conserved heat shock protein expressed in prokaryotic cells. Chaperone proteins are abundant and redundant in nature as they play fundamental functions for the maintenance of the cellular integrity. Indeed, they recognize and assist the folding of newly synthesized proteins, both preventing their aggregation and helping protein trafficking among different cellular compartments (Kim et al., 2013). Structurally DnaK (as well as the correspondent human counterpart family of heat shock proteins HSP70) is composed of two domains: a nucleotide binding domain (NBD) that binds adenosine-tri-phosphate (ATP) and a substrate binding domain (SBD), connected by a flexible linker (Genevaux et al., 2007). By an ATP-regulated process (ATPase activity) mediated by the NBD, the SBD binds a large repertoire of protein clients and confers chaperone function (Calloni et al., 2012) thus allowing the DnaK protein to recognize hydrophobic amino acid residues exposed by unfolded proteins and promote the *de novo* protein folding (Rüdiger et al., 1997). The binding of DnaK to client protein is transient and is driven by two classes of co-chaperones: DnaJ and the nucleotide exchange factor (NEF) (Bracher and Verghese, 2015). DnaJ stimulates the ATPase activity of DnaK, while NEFs promote the exchange of DnaK-bound ADP with ATP. Bacteria, mitochondria and chloroplasts have only one type of NEF called GrpE. In contrast, a large diversity of HSP70 NEFs has been discovered in the eukaryotic cells (Bracher and Verghese, 2015). Although a functioning chaperone complex requires the co-chaperones, DnaK can bind client proteins with an affinity regulated by ATP (Mayer and Gierasch, 2019). Indeed, chaperone proteins have the common ability to bind numerous client proteins (Rüdiger et al., 1997; Van Durme et al., 2009; Mayer and Gierasch, 2019; Ryu et al., 2020). To this regard the proteome of DnaK was previously solved in *Escherichia coli* cells and 700 interacting bacterial proteins were identified (Calloni et al., 2012). It was demonstrated that *E. coli* DnaK enriched proteins included a wide range of proteins with dominant functions associated to bacterial DNA replication, recombination, repair, cell division and chromosome segregation (Calloni et al., 2012).

Though most *Mycoplasma* are extracellular, some are able to invade eukaryotic cells (Yavlovich et al., 2004; Curreli et al., 2021) and several groups (Lo et al., 1993; Baseman et al., 1995; Yavlovich et al., 2004; Hegde et al., 2014), including ours (Curreli et al., 2021), have shown that they can grow intracellularly, indicating that the host invasion contributes to *Mycoplasmas* persistence and pathogenesis. To this regard, *Mycoplasmas* have been associated with some human cancers (Huang et al., 2001), including prostate cancer (Barykova et al., 2011), oral cell carcinoma (Henrich et al., 2014) and non-Hodgkin’s lymphoma (NHL) in HIV-seropositive subjects (Ainsworth et al., 2001). Moreover, chromosomal alterations and phenotypic changes have previously been observed *in vitro* in mouse and human cells infected with *Mycoplasma fermentans* subtype *incognitus*, and these aberrations did eventually result in the acquisition of malignant properties, including loss of anchorage dependency, ability to form colonies in soft agar, and tumorigenicity in nude mice (Zhang et al., 2006; Jiang et al., 2008; Namiki et al., 2009). In addition, infections with several *Mycoplasmas* (*fermentans*, *arginini*, *hominis*, and *arthritidis*) inhibit p53 activity and cooperate with Ras in oncogenic transformation, though the responsible bacterial protein has not been identified (Logunov et al., 2008). These findings indicate that, in some cases, *Mycoplasmas* could facilitate tumorigenesis, though no direct carcinogenic role for any *Mycoplasmas* has been demonstrated *in vivo*. Moreover, we have previously shown by phylogenetic amino acid analysis that some other bacteria associated with human cancers (including certain *Mycoplasmas*, *H. pylori* and *F. nucleatum*) have highly related DnaKs, suggesting a possible common mechanism of cellular transformation (Zella et al., 2018). This suggests that *Mycoplasmas*, and perhaps certain other bacteria with closely related DnaK, may have oncogenic activity mediated by common mechanism(s) DnaK-related.

We previously demonstrated that infection with a *M. fermentans* strain isolated in our laboratory from cells from an HIV-seropositive patients promoted lymphoma in a mouse model and that its DnaK interacts with key cellular proteins, including USP10 and PARP1, to hamper essential pathways related to DNA repair and p53 functions (Zella et al., 2018; Benedetti et al., 2020a). We also analyzed Mycoplasma-infected cells showing that DnaK mRNA and DnaK protein are both mainly found in the cytosol (Curreli et al., 2021) and that, similar to eukaryotic HSP70 (Becker et al., 2002; Theriault et al., 2006), DnaK can be taken up by bystander cells (Zella et al., 2018). It is unclear how the uptake or the release of DnaK inside the cell can affect host functions, though it is clear that identifying the key cellular targets of this bacterial chaperone protein can provide useful information on mechanisms of cellular transformation and possibly uncover alterations of other important cellular pathways.

Here we describe our results based on a proteomics approach aimed at characterizing eukaryotic client proteins by using label-free quantitative proteomics. We identified eukaryotic proteins that bind Mycoplasma DnaK from five different human tumor cell lines, representative of different cancer types. It is worth noting that Mycoplasmas are found in the tumor microenvironment of four tissues, namely gastric, colon adenocarcinoma, lung and prostate cancer (Benedetti et al., 2020b). Furthermore, we selected the fifth cancer line, a neuroblastoma cell line, due to the increasingly important association between gut and brain (Cryan et al., 2019; Muller et al., 2020), and the hypothesis that microbial proteins released by the gut microbiota (which include Mycoplasmas) could influence brain functions and cancers (Mehrian-Shai et al., 2019). We found that Mycoplasma DnaK binds 520 cellular proteins that we characterized based on enrichment and protein-protein interaction analysis. This study is the first to show how a bacteria chaperone protein can affect cellular pathways and it creates a platform for further functional and *in vivo* studies.

## Materials and methods

### Cell culture, plasmid, and transfection

Gastric adenocarcinoma cells AGS and Prostate adenocarcinoma cells PC3 were growth in F-12K medium (Kaighn’s Modification of Ham’s F-12 medium). Small cell lung cancer H446 were grown in RPMI-1640 medium. Neuroblastoma SH-SY5Y cells were grown in a 1:1 mixture of Eagle’s Minimum Essential medium (EMEM) and F12 medium. Colorectal carcinoma cells HCT116 were grown in McCoy’s 5a Medium Modified. All cell lines were purchased from the American Tissue Culture Collection (ATCC, Manassas, VA). The growth media were supplemented with 10% of fetal bovine serum (FBS; Thermo Fisher Scientific, Waltham, MA), 100 U/ml of penicillin, and 100 μg/ml of streptomycin (both from Thermo Fisher Scientific, Waltham, MA). The cells were cultured at 37°C in a humidified atmosphere with 5% CO_2_.

The full length DnaK from *M. fermentans* cloned into pcDNA 3.1 Directional/V5-His TOPO vector was previously described (Zella et al., 2018). A pcDNA 3.1/V5-His empty vector was used as a control in the transfection experiments. Transient transfection of the cells was performed with lipofectamine 2000 (Thermo Fisher Scientific, Waltham, MA – #11668) or Fugene HD (Promega, Madison, WI – #E2311), depending on the cell type. Lipofectamine 2000 was used to transfect HCT116 cells. Briefly, three T75 flasks were grown at 80% confluence. Transfection was performed in a mixture containing OptiMEM media (Thermo Fisher Scientific, Waltham, MA, USA), the plasmid DNA containing DnaK-V5-His or without the insert (control) and lipofectamine 2000 and incubated overnight. Two T75 were transfected with DnaK, and 1 T75 was transfected with the control empty vector. Transfected cells were harvested after 48 h. Transfections of AGS, PC3, H446 and SH-SY5Y were performed with Fugene HD. The protocol was similar to the one used for transfection with lipofectamine, with the difference that the mixture containing OptiMEM media (Thermo Fisher Scientific, Waltham, MA), the plasmid DNA containing DnaK-V5-His or without the insert (control) and Fugene HD reagent, were not removed from the cell culture media.

### Western blotting

Western blot was performed to verify that the transfection and the immunoprecipitation (IP) were successful and to validate Mass Spectrometry results. When Western blotting was used to verify the effectiveness of the transfection or of the immunoprecipitation, the membranes were probed with a mouse mAb against V5 tag (Thermo Fisher Scientific, Waltham, MA – #R960-25, dilution used 1:1,000) and a mouse mAb against β-actin (Cell Signaling Technology, Danvers, MA – #3700, dilution used 1:1,000). To validate proteins identified by Mass Spectrometry the following antibodies were used in the co-IP experiments: anti-PARP1 (R&D Systems, Minneapolis, MN – #AF-600, concentration used 0.4 μg/ml), anti-KU70 (Cell Signaling Technology, Danvers, MA – #4588, dilution used 1:1,000), anti-KU80 (Cell Signaling Technology, Danvers, MA – #2180, dilution used 1:1,000), anti-LIG3 (Thermo Fisher Scientific, Waltham, MA – #ma1-23191, dilution used 1:500-1:3,000), anti-β-catenin (Cell Signaling Technology, Danvers, MA – #8480, dilution used 1:1,000), anti-SF3B1 (Thermo Fisher Scientific, Waltham, MA – #PA541723, concentration used 1 μg/ml), anti-XRCC1 (Thermo Fisher Scientific, Waltham, MA – #MA1-12640, concentration used 1-2 μg/ml), anti-RPA/p70 (Santa Cruz Biotechnology, Dallas, TX – #SC-28304, dilution used 1:200), anti-DNA-PK (Cell Signaling Technology, Danvers, MA – #12311, dilution used 1:1,000) anti-DHX9 (Abcam, Cambridge, UK – ab26271, concentration used 1 μg/ml), anti-RUVBL2 (Novus Biologicals, Littleton, CO – #NBP2-01764, dilution used 1:500-2,000).

For Western blot analysis, cell monolayers were washed in cold PBS, detached using a scraper, and resuspended in RIPA lysis buffer (Sigma-Aldrich, St. Louis, MO, USA) in the presence of protease inhibitors (Sigma-Aldrich, St. Louis, MO, USA). The protein concentration was measured by the Bradford assay (Bio-Rad, Hercules, CA, USA). Thirty micrograms of protein were resolved by SDS/PAGE, transferred to a polyvinylidene difluoride (PVDF) membrane using trans-blot turbo transfer system (Bio-Rad), blocked in 5% non-fat dried milk in Tris-Buffered Saline (TBS) and probed overnight with the primary antibody. Blots were incubated with secondary HRP-conjugated antibodies (anti-rabbit IgG, Cell Signaling Technology, Danvers, MA – #7074, dilution used 1:1,000; anti-mouse IgG, Cell Signaling Technology, Danvers, MA – #7076, dilution used 1:1,000), developed using an ECL chemiluminescent substrate kit (Amersham Bioscience, Amersham, United Kingdom), and exposed and acquired using the ChemiDoc MP digital image system (Bio-Rad, Hercules, CA, USA).

### Immunoprecipitation

DnaK-V5 transfected cells were harvested at 48 h. After washing the monolayer with cold PBS, cells were detached with a scraper and resuspended in 1 ml of radioimmune precipitation buffer (Cell Signaling Technology, Danvers, MA) containing protease inhibitors (Sigma-Aldrich, St. Louis, MO, USA). Lysates were precleared with 50 μl of Dynabeads magnetic beads for 1 h at 4°C with end-over-end rotation. Non-specifically bound proteins were removed by a magnet. Next, 400 μg of lysate were incubated for 2 h at 4°C with end-over-end rotation with antibody-coated beads by using the IP Dynabeads Protein G Immunoprecipitation Kit (Thermo Fisher Scientific, Waltham, MA – #10007D). Specifically, for each analyzed cell line, four experimental IPs were performed including: a duplicate IP with anti-rabbit V5 Tag antibody (Abcam, Cambridge, United Kingdom – #ab9116, 1.775 mg/ml), one IP with the isotype control rabbit IgG (Abcam, Cambridge, United Kingdom – #ab172730, 1 mg/ml), one IP with anti-rabbit V5 Tag antibody that was pre-incubated with V5 blocking peptide (Sigma-Aldrich, St. Louis, MO – #V7754, 50 μg in the blocking mix). The beads with the bound proteins were collected with a magnet, washed three times with washing buffer and resuspended in precipitation buffer (provided with the kit). Following 10 min incubation at 70°C, the beads were removed from the proteins and resuspended in SDS-PAGE sample buffer. 1/6 of the total immuno-precipitated product was analyzed by SDS-PAGE immunoblot to verify that the IP procedure was successful, while the remaining material was analyzed by Mass Spectrometry.

### Liquid chromatography mass spectrometry sample analysis

The gel pieces from the band (2 large slice fractions per sample) were transferred to a siliconized tube and washed in 200 μl 50% methanol. The gel pieces were dehydrated in acetonitrile, rehydrated in 30 μl of 10 mM dithiolthreitol in 0.1 M ammonium bicarbonate and reduced at room temperature for 0.5 h. The DTT solution was removed and the sample alkylated in 30 μl 50 mM iodoacetamide in 0.1 M ammonium bicarbonate at room temperature for 0.5 h. The reagent was removed, and the gel pieces were dehydrated in 100 μl acetonitrile. Next, the acetonitrile was removed, and the gel pieces rehydrated in 100 μl 0.1 M ammonium bicarbonate. The pieces were dehydrated in 100 μl acetonitrile, the acetonitrile was removed, and the pieces completely dried by vacuum centrifugation. The gel pieces were rehydrated in 20 ng/μl trypsin in 50 mM ammonium bicarbonate on ice for 30 min. Any excess enzyme solution was removed and 20 μl 50 mM ammonium bicarbonate added. The sample was digested overnight at 37*^o^*C and the peptides formed extracted from the polyacrylamide in a 100 μl aliquot of 50% acetonitrile/5% formic acid. This extract was evaporated to 15 μl for MS analysis. The Liquid chromatography mass spectrometry (LC-MS) system consisted of a Thermo Electron Q Exactive HF mass spectrometer system with an Easy Spray ion source connected to a Thermo 75 μmx 15 cmC18Easy Spray column (through pre-column). 7 μl of the extract was injected and the peptides eluted from the column by an acetonitrile/0.1 M acetic acid gradient at a flow rate of 0.3 μl/min over 2.0 h. The nanospray ion source was operated at 1.9 kV. The digest was analyzed using the rapid switching capability of the instrument acquiring a full scan mass spectrum to determine peptide molecular weights followed by product ion spectra (Top10 HCD) to determine amino acid sequence in sequential scans. This mode of analysis produces approximately 25,000 MS/MS spectra of ions ranging in abundance over several orders of magnitude.

### Mass spectrometry data analysis

The data generated from the MS samples were analyzed by database search by using the Sequest search algorithm within Proteome Discoverer 2.4.1 (Thermo Fisher Scientific) against Uniprot Human Proteome database The UniProt Consortium (2017). The Sequest search results were uploaded into the Scaffold 4.11.0 software program (Proteome Software, Inc.) for quantitative data analysis. To ensure the quality of our approach we applied multiple controls and filtering conditions at different stages of the experiments, starting from duplicate experiments performed for each analyzed cell line. The following filters were applied for data analysis: 99% minimum protein ID probability, a minimum number of 2 unique peptides for one protein and a stringent minimum peptide ID probability of 95% (producing a FDR, 1%). The identified protein was removed from the list if detected in the control IPs with the isotype Ig or the anti-V5 Ig performed in the presence of V5 blocking peptide. To be included in the list, both duplicate samples needed to have a spectral count value. Spectral count values of 1 and a mean spectral count less than 5 were removed. Quantification was label free based upon total spectral counts. Duplicate spectral count list for each identified protein were generated for each cell line. One-way analysis of variance (ANOVA) with Benjamini Ochberg correction (Ferreira and Zwinderman, 2006) was performed with Scaffold software on normalized duplicate spectral counts of proteins differentially expressed between the five cell types, by selecting a p-value of significance ≤0.05 and a minimum spectral count value of 3.

### Surface plasmon resonance binding analysis of DnaK-DnaK and DnaK-PARP1

Surface plasmon resonance (SPR) binding studies of DnaK-DnaK and DnaK-PARP1 were performed at 25°C on a BIAcore T100 System (BIAcore, Inc., Piscataway, NY). The assay buffer was HBS-EP (10 mM HEPES, 150 mM NaCl, 0.05% surfactant P20, pH 7.4, 3 mM EDTA). DnaK (2274.9 RUs for DnaK-DnaK and 2220 RUs for DnaK-PARP1, respectively) was immobilized on CM5 sensor chips using the amine-coupling chemistry recommended by the manufacturer. Analytes were introduced into the flow cells at 35 μl/min in the running buffer. Association and dissociation were assessed for 120 s and 600 s, respectively for the DnaK-DnaK binding, while 250 s and 600 s, respectively for the DnaK-PARP1 binding analysis. Resonance signals were corrected for non-specific binding by subtracting the background of the control flow-cell. After each analysis, the sensor chip surfaces were regenerated with 10 mM glycine solution (pH 2.0) with MgCl 1M and equilibrated with the buffer before next injection.

### Gene enrichment analysis and enrichment clustering

Metascape was used to perform the enrichment analysis and the enrichment clustering (Zhou et al., 2019). The following ontology terms were included in the enrichment analysis: Gene Ontology (GO) Biological Processes (Ashburner et al., 2000), Reactome gene set (Fabregat et al., 2018), Kyoto Encyclopedia of Genes and Genomes (KEGG) Pathway (Kanehisa and Goto, 2000), Canonical pathways (Subramanian et al., 2005), Halmark Gene Set (Liberzon et al., 2015), CORUM (Ruepp et al., 2010) and WikiPathways (Martens et al., 2021). The enrichment analysis applied the hypergeometric statistical tests to identify input proteins that were significantly overexpressed, using a cutoff of 0.01 and an enrichment factor >1.5. To account for multiple testing the *q*-value was calculate by applying the Banjamini-Hochberg procedure (Ferreira and Zwinderman, 2006). The resulting ontology term were hierarchically clustered based on their similarity, by applying a kappa score >0.3 (Cohen, 1960). A cluster was represented by the most significant term. An enrichment network was also visualized with Cytoscape (V3.6.1) (Shannon et al., 2003), where each node represents a significant term, and the cluster was represented by terms with the same color. The nodes were linked by an edge based on their similarity score.

### Protein-protein interaction network

Metascape was used to assemble protein-protein interaction networks (Zhou et al., 2019). The bioinformatic program used Biogrid database (Oughtred et al., 2021) and applied the MCODE algorithm (Bader and Hogue, 2003) that combines the three most enriched ontology terms. The top 10 most significant MCODES were represented.

## Results

### Strategy for the identification of proteins binding DnaK transiently expressed in different cancer cell lines

We performed LC-MS analysis from five human cancer cell lines (HCT116, AGS, H446, PC3, SH-SY5Y) transfected with an expression vector transiently expressing *M. fermentans* DnaK-V5. Supplementary Figure 1 represents the experimental workflow.

**FIGURE 1:**
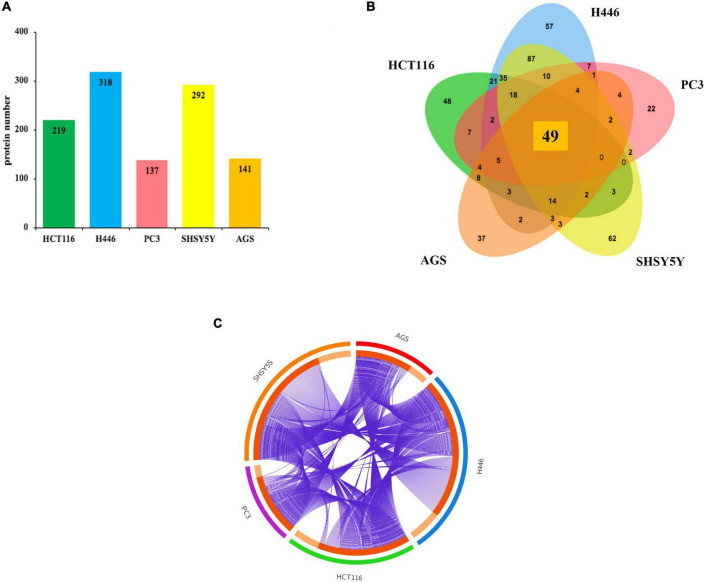
Lists the total number of proteins immunoprecipitated by DnaK-V5 and identified by mass spectrometry analysis in each cell type. **(A)** A total of 520 proteins were identified. The histogram indicates the number of hits for each cell type. **(B)** A Venn diagram (http://jvenn.toulouse.inra.fr/app/example.html) visualizes the number of shared and unique proteins obtained from each cell type. Of these, 49 proteins were shared among all five human cell lines analyzed. **(C)** The Circos plot (Krzywinski et al., 2009) shows the overlaps between the proteins from the input lists. On the outside, each arc represents the identity of the five analyzed cell lines (red for AGS, blue for H446, green for HCT116, purple for PC3, and orange for SH-SY5Y); on the inside, each arc represents a protein list, where each protein has a spot on the arc. The dark orange color of the inside arc represents the proteins that appear in multiple cell lines and the light orange color of the inside arc represents proteins that are unique to that cell type list. Purple lines link the proteins that are shared by multiple cell type. The greater the number of purple links and the longer are the inside dark orange arcs. This implies a greater overlap among the proteins from each cell line. A full list of the total 520 isolated proteins is given in Supplementary Table 1.

We identified 520 proteins across the five cell types (Figure 1 and Supplementary Table 1). The highest number of proteins was found in small cell lung carcinoma (*n* = 318) and neuroblastoma (*n* = 292), followed by the colorectal cancer (*n* = 219), gastric adenocarcinoma cells (*n* = 141) and finally prostate cancer (*n* = 137) (Figure 1A). A Venn diagram, used to visualize the number of shared and unique proteins obtained from each cell line, indicates that the shared hit in all five analyzed cell lines included 49 proteins (Figure 1B). Accordingly, the purple lines from the Circos plot link the proteins that are shared between the five cell lines, therefore showing the overlaps between proteins from different cell lines (Figure 1C). The detection of shared hits and overlaps among five different cancer cell types supports the reproducibility of our MS analysis. This finding also suggests that DnaK may have a higher binding affinity for these 49 eukaryotic proteins (listed in Supplementary Table 2), given that DnaK could recognize and bind these same 49 proteins from 5 different cell lines. However, due to the different nature of the 49 shared proteins, further studies will be necessary to understand the higher affinity of DnaK for these 49 proteins. On the other hand, we found that unique proteins can bind DnaK in each one of the 5 cell lines, as shown in the Venn diagram (Figure 1B) and indicated by the light orange inner arcs in the Circos plot (Figure 1C). This lack of concomitant expression could be due to different reasons, including tissue specificity, time-expression dependent, lack of sensitivity or differentiation/activation programs specific per each analyzed cell type. However, to simplify our analysis and gain a better understanding of the potential effects of these interactions, we decided to cluster together the proteins of each related pathway.

### Bioinformatics analysis for the enriched terms

Enrichment analysis showed that the list of proteins from the five cancer cell lines were overexpressed in 182 GO biological processes terms, 151 Reactome terms, 15 KEGG pathways, 62 CORUM and 7 WikiPathways terms (Supplementary Tables 1, 2). The top 20 most significantly enriched terms consisted with pathways related to RNA biological processes (mRNA processing, RNA metabolic process, RNA localization, RNA transport, mRNA translation, ribonucleoprotein complex subunit organization and gene silencing by miRNA, and mRNA splicing), DNA repair, chromatin biology (DNA-protein complexes, chromatin remodeling and chromosome organization) and cell cycle (Table 1).

**TABLE 1:** Top 20 significant enriched ontology terms in AGS, H446, PC3, HCT116, and SH-SY5Y according to the *p*-value and *q*-value.

GO	Category	Description	Count	%	Log10(P)	Log10(q)
R-HSA-8953854	Reactome Gene Sets	Metabolism of RNA	121	23.27	−83.36	−79
GO:0006402	GO Biological Processes	mRNA catabolic process	87	16.73	−68.75	−64.69
GO:0006412	GO Biological Processes	Translation	77	35.16	−65.12	−61.24
GO:1903311	GO Biological Processes	regulation of mRNA metabolic process	64	12.31	−44.85	−41.91
GO:0022618	GO Biological Processes	ribonucleoprotein complex assembly	57	10.96	−41.89	−39.04
GO:0006338	GO Biological Processes	chromatin remodeling	37	12.67	−36.4	−33.91
GO:0071103	GO Biological Processes	DNA conformation change	54	10.38	−34.64	−32.1
GO:0006281	GO Biological Processes	DNA repair	67	12.88	−33.37	−30.85
CORUM:1332	CORUM	Large Drosha complex	16	5.03	−27.65	−25.49
WP3888	WikiPathways	VEGFA-VEGFR2 Signaling Pathway	52	10	−26.09	−23.67
GO:0006403	GO Biological Processes	RNA localization	40	7.69	−26.01	−23.59
GO:0050684	GO Biological Processes	regulation of mRNA processing	32	6.15	−25.09	−22.68
R-HSA-73894	Reactome Gene Sets	DNA Repair	34	11.64	−22.86	−20.9
GO:0071824	GO Biological Processes	protein-DNA complex subunit organization	39	7.5	−21.95	−19.59
GO:0032200	GO Biological Processes	telomere organization	26	8.18	−20.92	−19
R-HSA-1640170	Reactome Gene Sets	Cell Cycle	57	10.96	−20.45	−18.12
GO:0072331	GO Biological Processes	signal transduction by p53 class mediator	36	6.92	−20.08	−17.76
GO:0051052	GO Biological Processes	regulation of DNA metabolic process	45	8.65	−19.84	−17.52
R-HSA-8878171	Reactome Gene Sets	Transcriptional regulation by RUNX1	34	6.54	−19.59	−17.28
GO:0006302	GO Biological Processes	double-strand break repair	29	9.12	−19.32	−17.46

Count = total number of proteins from the 5 cell lines proteomics list that are included in the corresponding onthology term; % = percentage of proteins from the 5 cell lines proteomics list that are found in the corresponding onthology term; Log10(P) = p-value in Log base 10; Log10(q) = multiple test adjusted p-value in Log base 10 by using the Banjamini-Hochberg procedure.

**FIGURE 2:**
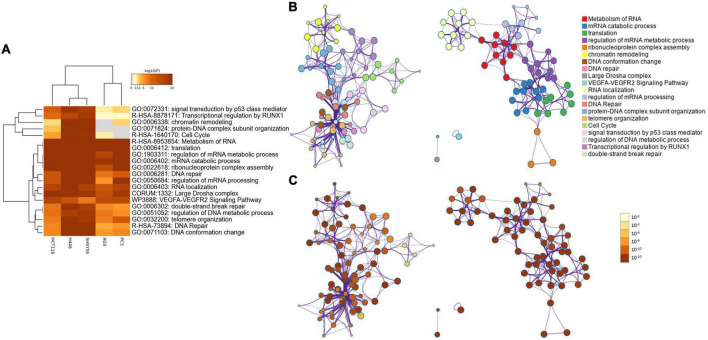
**(A)** Heatmap showing the top 20 enrichment terms across input proteins in the five cell lines, according to the *p*-value. **(B)** Network of enriched ontology clusters colored by cluster ID. A subset of enriched terms was selected based on the best p-values from each of the 20 clusters, and represented as a network plot, visualized by Cytoscape (Shannon et al., 2003). Each node represents an enriched term and is colored by its cluster ID. The 20 cluster IDs are listed on the side. **(C)** Network of enriched ontology clusters colored by *p*-value. The same network shown in **(B)** is represented colored by its p-value. On the side is the *p*-value scale ranging from 10^–2^ to 10^–20^.

The heatmap of the top 20 enriched terms (Figure 2A and Table 2) showed that in all the 5 cancer cell lines the ontology terms related to RNA biology were significantly enriched. Small cell lung cancer cells (H446) and neuroblastoma cells (SH-SY5Y) were hierarchically clustered together with a very high significant association for most of the terms. In contrast, the gastric (AGS) and prostate (PC3) adenocarcinoma cell lines clustered together and were not significantly enriched in the GO term of protein-DNA complex subunit organization and minimally or no significant in the GO term of chromatin remodeling and the Reactome term of cell cycle (Table 2 and Figure 2A).

**TABLE 2:** Top 20 significant clusters of the enriched ontology terms in AGS, H446, PC3, HCT116, and SH-SY5Y according to the *p*-value.

GO	Description	AGS Log10(P)	H446 Log10(P)	HCT116 Log10(P)	PC3 Log10(P)	SH-SY5Y Log10(P)
GO:0072331	signal transduction by p53 class mediator	−2.67	−19.46	−10.47	−3.49	−17.22
R-HSA-8878171	Transcriptional regulation by RUNX1	−2.15	−15.28	−9.14	−2.19	−18.30
GO:0006338	chromatin remodeling	0	−33.60	−3.26	−3.59	−36.40
GO:0071824	protein-DNA complex subunit organization	0	−19.72	−4.10	0	−21.90
R-HSA-1640170	Cell Cycle	−2.60	−19.79	−5.64	0	−19.56
R-HSA-8953854	Metabolism of RNA	−31.07	−73.48	−59.43	−40.64	−64.30
GO:0006412	translation	−20.69	−42.61	−65.12	−23.20	−31.58
GO:0006402	mRNA catabolic process	−19.05	−55.35	−65.18	−25.69	−47.06
GO:1903311	regulation of mRNA metabolic process	−15.37	−37.67	−33.44	−28.60	−34.46
GO:0022618	ribonucleoprotein complex assembly	−13.33	−26.73	−39.63	−23.90	−28.05
GO:0006281	DNA repair	−8.43	−33.06	−12.33	−10.36	−28.74
GO:0050684	regulation of mRNA processing	−6.28	−22.12	−12.48	−20.01	−20.36
GO:0006403	RNA localization	−12.03	−23.19	−15.66	−15.93	−16.29
CORUM:1332	Large Drosha complex	−13.43	−27.65	−20.36	−18.02	−18.84
WP3888	VEGFA-VEGFR2 Signaling Pathway	−14.29	−12.81	−15.99	−14.49	−8.16
GO:0006302	double-strand break repair	−6.98	−19.32	−8.48	−6.10	−17.10
GO:0051052	regulation of DNA metabolic process	−8.23	−16.98	−8.69	−6.59	−18.11
GO:0032200	telomere organization	−7.74	−20.92	−7.88	−11.47	−18.16
R-HSA-73894	DNA Repair	−5.14	−21.64	−7.17	−6.09	−22.86
GO:0071103	DNA conformation change	−6.15	−32.32	−7.38	−7.18	−31.48

Log10(P) = *p*-value in Log base 10.

A network representation of the enriched terms (Figure 2B) highlights 2 major subnetworks. One included multiple RNA biological processes while the other subnetwork included chromatin remodeling, DNA conformation change, DNA repair, protein-DNA complex subunit organization, telomere organization, cell cycle, signal transduction by p53, regulation of DNA metabolic process and transcriptional regulation of RUNX1. Two separated terms included the CORUM large Drosha complex and the WikiPathways of VEGFa-VEGFR2 signaling pathway. The networks colored by p-value (Figure 2C) highlights how the subnetwork including RNA-related processes was highly significant for all the terms, while the second subnetwork included some terms less significant (e.g., cell cycle and transcriptional regulation by RUNX1).

### Protein-protein interaction analysis

**FIGURE 3:**
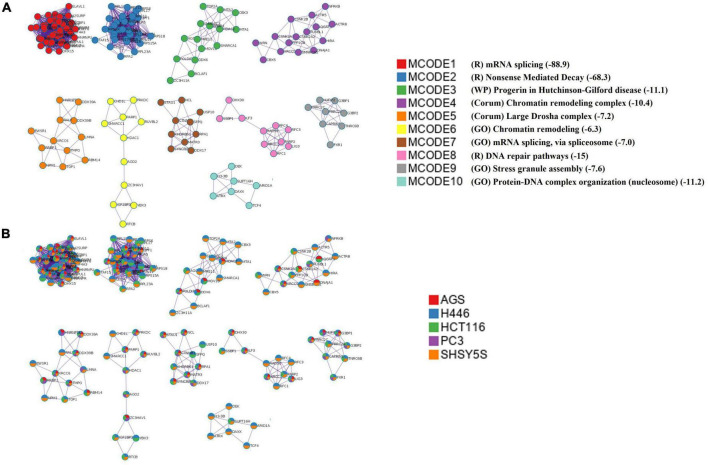
Top 10 significant protein-protein interaction networks. **(A)** The top 10 MCODE complexes formed by the 520 proteins from the pulled list of the 5 analyzed cell lines. The PPI included the mRNA splicing pathway (MCODE1 and 7), the nonsense-mediated decay (NMD) (MCODE2), the effect of progerin on the involved genes in Hutchinson-Gilford Progeria Syndrome (MCODE3), the INO80 chromatin remodeling complex (MCODE4), Large Drosha complex (MCODE5), chromatin remodeling complex (MCODE6), DNA repair pathways (MCODE8), stress granule assembly (MCODE9), and protein-DNA complex subunit organization (MCODE10). The mRNA splicing pathway was the most significant. *P*-value in Log 10 are given in parenthesis in the Legend and in Supplementary Table 3. In the Legend R = Reactome, WP = WikiPathways, GO = GO term. **(B)** The network nodes are displayed as pie. Color code for pie sector represents a protein list from each of the five analyzed cell lines. The keys codes for the MCODEs shown in this figure are listed in Supplementary Table 3, together with their correspondent *p*-value.

To better understand the interplay between the identified proteins, we performed a protein-protein interaction (PPI) network analysis by using Metascape (Zhou et al., 2019). Biologically relevant networks were obtained by applying a Molecular Complex Detection (MCODE) algorithm (Oughtred et al., 2021; Supplementary Table 3). Our data show that the most significant interaction networks covered by the proteins binding DnaK include the mRNA splicing network (Log10 *p*-value –88.9), the Nonsense-Mediated Decay (NMD) complex (Log10 *p*-value –68.3) and the DNA repair pathways (Log10 *p*-value –15) (Figure 3 and Supplementary Table 3).

**FIGURE 4:**
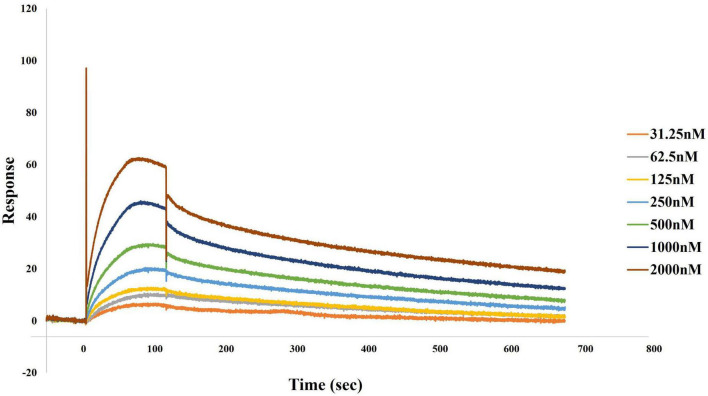
Direct binding of DnaK to DnaK (self-binding) as determined by surface plasmon resonance (SPR). Association of DnaK at different concentrations on 2,274.9 response units of DnaK immobilized on a CM5 biosensor chip proceeded at a flow rate of 35 μl/min for 120 s, followed by a 600 s-dissociation in HBS-EP. A preliminary kinetic analysis yielded a Kd value of 2.956e^–8^M.

Finally, we confirmed that DnaK forms homodimers, which are believed to represent a more efficient configuration of the protein in its interactions with cellular components. In fact, previous cross-linking experiments demonstrated that DnaK forms a transient dimer upon ATP binding. Biochemical analyses then showed that this dimer is essential for the efficient interaction of the protein with the co-chaperone molecule Hsp40, and in turn allows for efficient DnaK activity (Sarbeng et al., 2015). By using surface plasmon resonance binding analysis we indeed confirmed that DnaK used in our experiments could forms homodimers. Based on our data, kinetic analysis of DnaK-DnaK association yielded a Kd value of 2.956e^–8^M (Figure 4).

### Validation of the mass spectrometry identified proteins implicated in the reactome pathway of DNA repair (R-HSA-73894)

**FIGURE 5:**
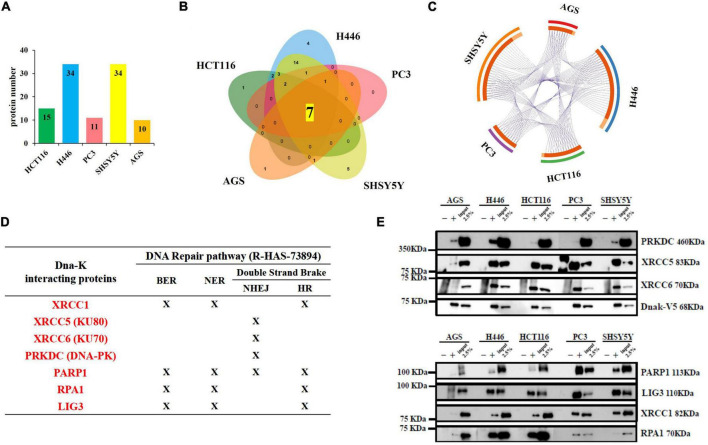
Lists of the total number of proteins immunoprecipitated by DnaK-V5 and identified by mass spectrometry analysis in each cell type that are part of the DNA repair Reactome pathway (R-HSA-73894). **(A)** A total of 34 proteins were identified (see Supplementary Tables 1, 2). The histogram indicates the number of hits for each cell type. **(B)** A Venn diagram visualizes the number of shared and unique proteins obtained from each cell type. Of these, 7 proteins were shared among all five human cell lines analyzed. **(C)** The Circos plot shows the overlaps between the proteins from theDNA repair pathway. On the outside, each arc represents the identity of the 5 cell lines (red for AGS, blue for H446, green for HCT116, purple for PC3 and orange for SH-SY5Y); on the inside, each arc represents a protein list, where each protein has a spot on the arc. The dark orange color of the inside arc represents the DNA repair proteins that appear in multiple lists and the light orange color of the inside arc represents DNA repair proteins that are unique to that cell type list. Purple lines link the proteins that are shared by multiple cell types. The greater the number of purple links and the longer are the dark orange arcs. This implies a greater overlap among the protein from each cell line. A full list of the total 34 isolated proteins from the DNA repair Reactome pathway is given in Supplementary Table 2. **(D)** Table indicating the membership of the seven proteins in the 4 DNA repair pathways of Base Excision Repair (BER), Nucleotide Excision Repair (NER), Non-Homologous End Joining (NHEJ) and Homologous Recombination (HR). **(E)** Western blot from DnaK immunoprecipitation comparing the five cancer cell lines. On the left side, the Molecular Weight (MW) Marker is indicated. On the right side, the name and MW of each validated protein are given. For each of the 5 cell types, three samples were loaded: the immunoprecipitated DnaK-transfected lysate with the antibody isotype control, the immunoprecipitated DnaK-transfected lysate with anti-V5 antibody and 2.5% load of the DnaK-transfected lysate used for the immunoprecipitations.

We previously demonstrated the effect of DnaK in decreasing p53 tumor-suppressor activity and its inhibitory effect on PARP1 (Zella et al., 2018). To confirm and expand our findings, we first performed enrichment analysis of both DNA repair pathways from the GO ontology term (GO:0006281) and the reactome (R-HAS-73894). The top 20 significant clusters in all 5 analyzed cell lines are showed in Table 2. Next, we sought to explore further the correlation of DnaK with the reactome pathway of the DNA repair (R-HSA-73894). The most abundant proteins were identified from the cell lines of small cell lung carcinoma and neuroblastoma (*n* = 34 for both cell lines) (Figure 5A). The Venn diagram indicates that the shared hits in all five analyzed cell lines included 7 proteins (Figure 5B) and the purple lines in the Circos plot visualize the shared hits (Figure 5C). Among these proteins (Figure 5D), X-Ray Repair Cross Complementing 5 (XRCC5), X-Ray Repair Cross Complementing 6 (XRCC6) and Protein Kinase, DNA-Activated, Catalytic Subunit (PRKDC) are essential components of the DNA double strand repair pathway of Non-Homologous End Joining (NHEJ) (Lieber, 2010). In this contest, the XRCC5-XRCC6 heterodimer plays a central role in binding to the ends of the double strand broken DNA and in recruiting PRKDC (Gottlieb and Jackson, 1993). PRKDC is a Serine/threonine-protein kinase that acts as a sensor of DNA damage and phosphorylates itself and a number of proteins participating in the NHEJ machinery, favoring and promoting the execution of the repair (Drouet et al., 2006).

The remaining shared hits in the 5 cancer cell lines included X-Ray Repair Cross Complementing 1 (XRCC1), PARP1, Replication Protein A1 (RPA1) and DNA ligase 3 (LIG3) (Figure 5D). These proteins are known player during multiple DNA repair processes including Base Excision Repair (BER) (Kutuzov et al., 2021), Nucleotide Excision Repair (NER) (Moser et al., 2007, Marteijn et al., 2014) and the double strand repair pathway of Homologous Recombination (HR) (Heyer et al., 2010) Specifically, PARP1 activity is necessary in different steps of the repair process. It is an essential sensor of the DNA damage (Ali et al., 2012) and its catalyzes the addiction of poly(ADP-ribose) (PAR) chains to recruit repair proteins (Jungmichel et al., 2013), including XRCC1 (Masson et al., 1998), in the DNA damage site. XRCC1 acts as a scaffold protein for the recruitment of factors involved in the repair (Caldecott, 2019), including LIG3 (Nash et al., 1997), that seals the nicked DNA ends (Tomkinson and Sallmyr, 2013). PARP1 has also shown to form a complex with PRKDC and catalyzes its PARylation, that is important for regulating PRKDC activity (Han et al., 2019). Binding of DnaK to these molecular players of the DNA repair pathways may impair the ability of the cell to recognize the damaged DNA and execute the repair, possibly leading to genome instability.

Duplicate spectral counting show that XRCC5, XRCC6 and PARP1 (Supplementary Figure 2) where recovered from the IP with high spectral count values (around 200 hits) suggesting they were more abundant in the DnaK’s immune-precipitates, while the remaining 4 proteins had less hits. The spectral count of these proteins varied considerably in the five analyzed cell lines and were more abundant in the small cell lung cancer H446 and the neuroblastoma cells SH-SY5Y and constantly reduced in the gastric adenocarcinoma AGS (Supplementary Figure 2). Validation analysis was carried out by western blot analysis of DnaK immune-precipitates (Figure 5E). The common proteins identified by Mass Spectrometry were detected in all the five cancer cell lines, even if at different levels. Of note, the absence of a band in the IP with the IgG control demonstrates the specificity of the binding (Figure 5E). The binding affinity of DnaK to PARP1 was also validated by Surface Plasmon Resonance (SPR) equilibrium analysis, and preliminary kinetic analysis yielded a Kd value of <25 nM (2.501e^–8^M) (Supplementary Figure 3).

### Western blot validation of the mass spectrometry identified proteins implicated is the top enriched terms of RNA metabolism, chromatin remodeling and translation

**FIGURE 6:**
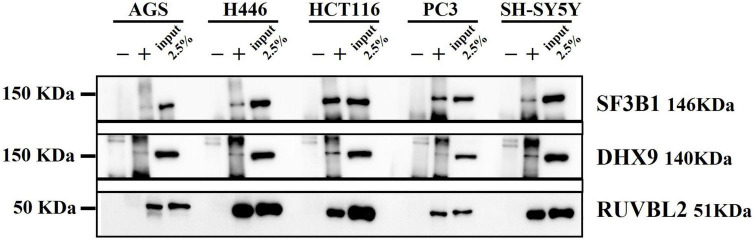
Western blot from DnaK immunoprecipitation comparing the five cancer cell lines. On the left side the Molecular Weight (MW) Marker is indicated. On the right side the name and MW of each validated protein are given. For each of the five cell types, three samples were loaded: the immunoprecipitated DnaK-transfected lysate with the antibody isotype control, the immunoprecipitated DnaK-transfected lysate with anti-V5 antibody and 2.5% load of the Dank-transfected lysate used for the immunoprecipitations.

To validate the Mass Spectrometry identified proteins enriched in the top pathways, we performed Western Blot to identify DnaK-bound immunoprecipitated proteins (Figure 6). Splicing Factor 3B subunit 1 (SF3B1), a component of the SF3B complex (Cretu et al., 2016) involved in pre-mRNA splicing (Supplementary Table 4), was confirmed as a representative factor of the mRNA splicing term (R-HSA-72163). DExH-box helicase 9 (DHX9), a multifunctional nucleic acid helicase (Jain et al., 2010), was validated as a component of various ontology terms including RNA metabolism (R-HSA-8953854), translation (GO:0006412), RNA-mediated gene silencing (CORUM: 1332), as well as DNA repair (R-HSA-73894) (Supplementary Table 4). In addition, we validated RuvB-like AAA ATPase-2 (RUVBL2), an enzyme with ATPase and helicase activity (Puri et al., 2007; Figure 6). RUVBL2, together with RUVBL1, is a component of various chromatin-remodeling complexes including the Nucleosome Acetyltransferase of Histone 4 (NuA4) histone acetyltransferase complex (Jha et al., 2008). By forming complexes, RUBVL2 regulates the accessibility of the DNA to proteins and therefore plays a critical role in major pathways related to chromatin remodeling, DNA damage response, ribonucleoproteins assembly (GO:0022618), protein-DNA complex subunit organization (GO:0071824) and cell cycle (R-HAS-1640170) (Supplementary Table 4; Jha and Dutta, 2009).

## Discussion

Some species of Mycoplasmas, including *M. fermentans*, are associated with cancers (Ainsworth et al., 2001; Barykova et al., 2011; Henrich et al., 2014) and we previously demonstrated that Mycoplasma infection promoted lymphoma in a mouse model and its DnaK, a chaperone protein belonging to the HSP70 family, interacts with key cellular proteins to hamper essential pathways related to DNA repair and p53 functions (Zella et al., 2018). Chaperone proteins are fundamental for a correct protein folding, prevent a protein aggregation, and help to remove misfolded and incomplete proteins (Saibil, 2013). Since Mycoplasmas are able to invade eukaryotic cells (Lo et al., 1993; Baseman et al., 1995; Yavlovich et al., 2004; Hegde et al., 2014; Curreli et al., 2021), the release of bacteria components, including DnaK, inside the host cell is likely to contribute to Mycoplasma’s pathogenesis and interfere with the maintenance of the normal cell functions. Indeed, we previously demonstrated that upon invasion, DnaK protein is found inside the infected cells (Curreli et al., 2021) and that DnaK is taken up by bystander cells (Zella et al., 2018).

This work stems from our previous efforts in understanding how Mycoplasma DnaK is able to interfere with important cellular pathways. To this regard, we previously demonstrated that DnaK binds to PARP1 and USP10, affecting DNA repair and p53-dependent activities (Zella et al., 2018; Benedetti et al., 2020a). Here we deepen our investigation of DnaK’s key cellular targets to gain information about the possible role of DnaK in promoting cellular transformation and altering important cellular functions. For this purpose, we employed a label-free quantitative proteomics approach to identify the eukaryotic proteins binding Mycoplasma’s DnaK, upon transient transfection in 5 different human cancer cell lines. We found that DnaK interacts with a total of 520 proteins and their amount number ranges from 137 to 318 depending on the cell line. Our MS analysis showed that the shared hits in all 5 analyzed cell lines included 49 proteins, indicating conserved key target proteins among the analyzed cancer cell lines. On the other hand, the finding of unique proteins binding DnaK in the different cell lines is probably reflective of a cell-specific differentiation program.

The ability to bind a wide array of proteins is a common feature of chaperone proteins (Calloni et al., 2012; Ryu et al., 2020), and 700 interacting proteins were previously isolated from the proteomics analysis of DnaK in *E. coli* (Calloni et al., 2012). Similarly, the proteome of HSP70 and HSC70 (Heat shock cognate 71 kDa protein) was solved in eukaryotic cells and approximately 772 potential interactors were identified (Ryu et al., 2020). Indeed binding of HSP70s to their client proteins have been defined a “selectively promiscuous” process (Mayer and Gierasch, 2019) since chaperones can bind hydrophobic amino acid residues exposed by many unfolded proteins, but not all the proteins in the proteome (Rüdiger et al., 1997). Our current findings that a bacteria chaperone can bind numerous eukaryotic proteins suggest that the previously reported 57% shared sequence similarity (Chiappori et al., 2015) between DnaK and the most common human equivalent HSC70/HSP72 (Kim et al., 2013) might be relevant for the binding. Indeed, members of the HSP70 family of chaperones are involved in some of the most critical functions of the proteostasis network (Mayer and Gierasch, 2019). Our data thus further support the theory that the mechanism of binding proteins by prokaryotic and eukaryotic chaperones has remained evolutionary conserved (Mayer and Gierasch, 2019). To this regard, we are investigating whether the group of identified bacteria-DnaK-interacting human proteins has a significant overlap with the proteins interacting with human HSP70, and the effect of DnaK binding on these client proteins. This could indeed provide with a better understanding of the full scale of potential disruption of cellular pathways that DnaK could promote.

The activity of HSP70/DnaK is regulated by cycles of ATP binding and hydrolysis (Calloni et al., 2012; Nunes et al., 2015), and this in turn allows DnaK interaction with client proteins with an affinity modulated by the ATP itself (Mayer and Gierasch, 2019). However, DnaK has a weak ATPase activity, and like other chaperone proteins, it needs to cooperate with co-chaperones and cellular factors to accelerate ATPase activity and eventually accomplish its proper function (Bracher and Verghese, 2015; Clerico et al., 2015). Since in our experimental system only the bacterial DnaK was expressed, without the respective co-chaperones DnaJ and NEF (Bracher and Verghese, 2015), we evaluated potential partner proteins that could support its functional status. We showed that DnaK binds the protein disulfide isomerase family A member 6 (PDIA6) in all the analyzed cell lines, that is a member of the protein disulfide isomerase (PDI) family (Ellgaard and Ruddock, 2005) with a chaperone activity (Kikuchi et al., 2002). We also found that in the gastric adenocarcinoma cells AGS and neuroblastoma SH-SY5Y cells DnaK binds DnaJA1, a member of the HSP40 family of molecular co-chaperones (Qiu et al., 2006). DnaJA1 is well known for regulating eukaryotic HSP70 by stimulating ATPase activity thus generating the ADP-bound that interacts stably with the substrates (Qiu et al., 2006). In addition, we showed that DnaK forms homodimers. According to previously published data (Sarbeng et al., 2015), this homodimeric structure is considered more efficient in its ability to control the proper folding of the client proteins. Based on our data, it is thus very likely that Mycoplasma DnaK could indeed exerts its activity inside the eukaryotic host cell by forming homodimers and hijacking cellular co-chaperones like DnaJA1. Further studies are needed to assess the extent and the biological significance of such bindings.

Enrichment analysis revealed a sub-network which comprised ontology terms related to DNA-proteins interactions and included chromatin remodeling, DNA conformation change, DNA repair, protein-DNA complex subunit organization, telomere organization, cell cycle, signal transduction by p53, regulation of DNA metabolic process and transcriptional regulation of RUNX1. All these listed terms are crucial for the maintenance of genomic stability and DNA organization. We choose some of the most important proteins, namely DnaK-binding proteins XRCC5, XRCC6 and PRKDC, known to participate in NHEJ repair process (Lieber, 2010), for by Western blot analysis validation. Similarly, we validated XRCC1, PARP1, RPA1 and LIG3, known molecular participants in the DNA repair processes of BER (Kutuzov et al., 2021), NER (Moser et al., 2007; Marteijn et al., 2014) and HR (Heyer et al., 2010). Binding of DnaK to these regulators of multiple DNA repair pathways may impair the ability of the cell to recognize the damaged DNA and execute the repair, possibly leading to genome instability and predispose the cell to transformation. These data support our previous finding showing that infection of SCID mice with a strain of *M. fermentans* promotes lymphomagenesis, further indicating that interaction of DnaK with proteins involved in DNA repair pathways may have a role in cellular transformation (Zella et al., 2018).

The other major sub-network enriched was comprised of RNA biological processes, including RNA metabolism, mRNA catabolic process, mRNA splicing, nonsense-mediated decay, ranked highest among the ontology terms significant from the list. Those interactions were unexpected since, in contrast to prokaryotes, bacteria do not possess a spliceosome. These findings imply that DnaK could be directly associated with mRNA splicing/processing complexes. This is in agreement with the finding that the eukaryotic HSP70 family member binds and stabilizes a target mRNA independently from its chaperone activity (Kishor et al., 2017). It is also in agreement with a previous study demonstrating that DnaK from *E. coli* can bind AU rich RNAs and that the co-chaperones DnaJ and GrpE can modulate this effect (Zimmer et al., 2001). Consistent with these findings, the implication of DnaK in multiple processes of eukaryotic RNA biology may reduce the performance of the cellular quality control machinery or may interfere with the efficiency of protein translation in the cells infected with the bacteria.

Earlier studies have used proteomics-based analysis to identify networks of protein-protein interaction in several *in vitro* models of infection with the whole infectious agent (Urwyler et al., 2009; Noster et al., 2019; Noto et al., 2019). Our previous observation that binding of DnaK to the target proteins has an inhibitory effect with their function (Zella et al., 2018; Benedetti et al., 2020a) clearly indicates that the intrusion of a bacteria chaperone in the eukaryotic cells may compete with the activity of the eukaryotic chaperones and inhibit the function of the bound proteins. For this reason, we decided to focus our proteomic analysis around DnaK. Protein-protein interaction network analysis indicated that the most significant biological complexes formed by the proteins binding DnaK include: the mRNA splicing network (Log10 *p*-value –88.9), the Nonsense-Mediated Decay (NMD) complex (Log10 *p*-value –68.3) and the DNA repair pathways (Log10 *p*-value –15).

Here we show for the first time that a bacteria chaperone protein interacts with critical components of important cellular pathways in certain human cancer cell lines. Future studies are needed to broaden our analysis to include the specific interactions of DnaK with proteins from different cell types, both of normal and transformed phenotype. Further studies are also needed to both assess the biological outcomes of such interactions and to elucidate the mechanism(s) responsible for any change in client proteins function, whether is through simply direct binding to DnaK or improper folding. This would allow us to better understand the extent of DnaK interference with a number of cellular pathways active at different stages of cell development and differentiation. In addition, the nature of the binding of bacteria DnaK with eukaryotic proteins needs to be investigated. For example, we don’t know whether the DnaK-client’s bindings are transient, as normally occurs for chaperone proteins (Bracher and Verghese, 2015) or stable, and we don’t know the fate or the effect on the activity of the client proteins bound by DnaK.

Overall, the role of *Mycoplasma fermentans* in human diseases, including cancer, has been highlighted by several studies, and this research, together with other previous data (Benedetti et al., 2020b,Benedetti et al., 2021), strongly indicate that this bacterium and others expressing DnaKs similar in amino acid composition and structure may play a more complex role beyond the one of being simple opportunistic bacteria. Given the close interactions between bacteria and host cells in the local microenvironment (Maman and Witz, 2018), these data should help provide the foundation for future mechanistic studies on how bacteria interfere with essential cellular processes.

## Data availability statement

The raw mass spectrometric data files presented in the study are deposited in the PRIDE repository, with the accession number PXD035477 (http://www.ebi.ac.uk/pride/archive/projects/PXD035477).

## Author contributions

SC, RCG, and DZ: conceptualization. SC and DZ: data curation. SC: formal analysis. RCG and DZ: funding acquisition. SC, FB, AM, FC, and DZ: investigation. SC and WY: methodology. SC, RCG, and DZ: supervision. SC, FB, AM, and FC: visualization. NS: mass spectrometry. SC: writing – original draft. SC, FB, WY, AM, FC, RCG, NS, and DZ: writing – review and editing. All authors contributed to the article and approved the submitted version.

## Supplementary material

SUPPLEMENTARY FIGURE 1 Experimental workflow. **(A)** Five human cancer cell lines were transfected with DnaK-V5 expression vector. **(B)** Proteins were extracted in non-denaturing conditions and DnaK-V5 expression was validated by Western Blot (WB). **(C)** Precleared lysates were separated in 4 parts to perform immunoprecipitation (IP): 1. IP with immunoglobulin (Ig) isotype control, 2. Duplicate IP with anti-V5 Ig, 3. IP with anti-V5 Ig in the presence of V5 blocking peptide. **(D)** All IP were confirmed by WB and **(E)** samples were tryptic digested and subjected to Mass Spectrometry (MS) for protein identification. **(F)** The identified proteins were analyzed using the Scaffold proteome Software program. **(G)** Further data cleaning was applied to select only proteins commonly present in the duplicate samples, while proteins pulled from the IP with the isotype Ig and the anti-V5 Ig in the presence of V5 blocking peptide were subtracted.

SUPPLEMENTARY FIGURE 2 Quantitative comparison of DnaK-V5-binding proteins and detected by Mass Spectrometry analysis in the five cancer cell lines. Duplicates MS/MS data of proteins binding DnaK-V5 from five cancer cell lines was quantitatively determined by spectra counting in Scaffold software. PARP1, RPA1, LIG3, XRCC1, XRCC5, XRCC6, and PRKDC were chosen to represent common proteins in the 5 cell lines and components of the Reactome pathway of DNA repair (R-HSA-73894). ANOVA statistical analysis with Benjamini Ochberg correction (Ferreira and Zwinderman, 2006), was performed with Scaffold 4 on normalized duplicate spectra counts of proteins differentially expressed between the five cell types, by selecting a *p*-value of significance <0.05.

SUPPLEMENTARY FIGURE 3 Direct binding of PARP1 to DnaK as determined by surface plasmon resonance (SPR). Association of PARP1 at different concentrations on 2,220 response units of DnaK immobilized on a CM5 biosensor chip proceeded at a flow rate of 35 μl/min for 250 s, followed by a 600 s-dissociation in HBS-EP buffer. A preliminary kinetic analysis yielded a Kd value of <25 nM (2.501e^−8^M).

## Abbreviations

PARP1: Poly (ADP-Ribose) Polymerase-1
USP10: Ubiquitin carboxyl-terminal hydrolase 10
LS-MC: Liquid Chromatography Mass Spectrometry.
