# Supplementary material for: Characterization of the interactome profiling of Mycoplasma fermentans DnaK in cancer cells reveals interference with key cellular pathways
Source: Front Microbiol. 2022 Oct 28;13:1022704. doi: 10.3389/fmicb.2022.1022704 (PMC9651203; doi:10.3389/fmicb.2022.1022704)
Supplement: Supplementary file 1 [file Data_Sheet_1.ZIP › Supplemental/Table S2.pdf]

**Table S2: Total spectra counts of the 49 shared duplicates proteins from DnaK-V5 immunoprecipitation in transfected AGS, H446, HCT116, PC3 and SH-SY5Y cancer cell lines.**

| | | | | | | Total Spectrum Count | | | | | | | | | | STANDARD DEVIATIONS | | | | |
|---|---|---|---|---|---|---|---|---|---|---|---|---|---|---|---|---|---|---|---|---|
| | | | | | | AGS | | H446 | | HCT116 | | PC3 | | SH-SY5Y | | AGS | H446 | HCT116 | PC3 | SH-SY5Y |
| **Identified Shared Proteins (49)** | **Accession Number** | **Alternate ID** | **MW** | **ANOVA Test (p-value): *(p < 0.00832)** | **Quantitative Profile** | **IP1** | **IP2** | **IP1** | **IP2** | **IP1** | **IP2** | **IP1** | **IP2** | **IP1** | **IP2** | | | | | |
| X-ray repair cross-complementing protein 6 | P12956 | XRCC6 | 70 kDa | 0.42 | [] | 110 | 100 | 207 | 213 | 115 | 209 | 60 | 171 | 224 | 147 | 7.07 | 4.24 | 66.47 | 78.49 | 54.45 |
| X-ray repair cross-complementing protein 5 | P13010 | XRCC5 | 83 kDa | 0.36 | [] | 120 | 99 | 247 | 236 | 111 | 201 | 66 | 179 | 290 | 140 | 14.85 | 7.78 | 63.64 | 79.90 | 106.07 |
| Poly [ADP-ribose] polymerase 1 | P09874 | PARP1 | 113 kDa | 0.0036 | AGS high, H446 low, HCT116 high, PC3 high, SHSY5S high | 129 | 108 | 55 | 44 | 89 | 200 | 106 | 165 | 189 | 142 | 14.85 | 7.78 | 78.49 | 41.72 | 33.23 |
| Splicing factor 3B subunit 1 | O75533 | SF3B1 | 146 kDa | 0.41 | [] | 51 | 55 | 95 | 87 | 34 | 124 | 49 | 63 | 70 | 67 | 2.83 | 5.66 | 63.64 | 9.90 | 2.12 |
| Polyadenylate-binding protein 1 | P11940 | PABPC1 | 71 kDa | 0.42 | [] | 35 | 40 | 57 | 56 | 49 | 81 | 33 | 50 | 59 | 43 | 3.54 | 0.71 | 22.63 | 12.02 | 11.31 |
| DNA-dependent protein kinase catalytic subunit | P78527 | PRKDC | 469 kDa | 0.018 | [] | 75 | 43 | 45 | 40 | 18 | 39 | 15 | 29 | 65 | 51 | 22.63 | 3.54 | 14.85 | 9.90 | 9.90 |
| RuvB-like 2 | Q9Y230 | RUVBL2 | 51 kDa | 0.032 | [] | 8 | 11 | 40 | 42 | 45 | 27 | 42 | 72 | 103 | 82 | 2.12 | 1.41 | 12.73 | 21.21 | 14.85 |
| General vesicular transport factor p115 | O60763 | USO1 | 108 kDa | 0.12 | [] | 48 | 44 | 32 | 31 | 37 | 102 | 45 | 35 | 56 | 50 | 2.83 | 0.71 | 45.96 | 7.07 | 4.24 |
| Protein disulfide-isomerase A6 | Q15084 (+4) | PDIA6 | 48 kDa | 0.63 | [] | 60 | 46 | 47 | 53 | 34 | 58 | 36 | 56 | 93 | 41 | 9.90 | 4.24 | 16.97 | 14.14 | 36.77 |
| Splicing factor 3B subunit 3 | Q15393 | SF3B3 | 136 kDa | 0.63 | [] | 30 | 21 | 58 | 45 | 17 | 91 | 28 | 30 | 54 | 38 | 6.36 | 9.19 | 52.33 | 1.41 | 11.31 |
| DNA ligase 3 | P49916 (+1) | LIG3 | 113 kDa | 0.0033 | AGS low, H446 low, HCT116 low, PC3 low, SHSY5S high | 20 | 16 | 38 | 32 | 15 | 39 | 13 | 24 | 100 | 67 | 2.83 | 4.24 | 16.97 | 7.78 | 23.33 |
| Probable ATP-dependent RNA helicase DDX5 | P17844 | DDX5 | 69 kDa | 0.33 | [] | 32 | 27 | 37 | 34 | 28 | 43 | 23 | 25 | 32 | 24 | 3.54 | 2.12 | 10.61 | 1.41 | 5.66 |
| Ribonucleoprotein PTB-binding 1 | A0A087WZ13 (+1) | RAVER1 | 78 kDa | 0.25 | [] | 39 | 33 | 55 | 62 | 17 | 46 | 24 | 56 | 49 | 29 | 4.24 | 4.95 | 20.51 | 22.63 | 14.14 |
| Protein transport protein sec16 | J3KNL6 | SEC16A | 252 kDa | 0.02 | [] | 21 | 16 | 24 | 22 | 11 | 51 | 19 | 33 | 67 | 52 | 3.54 | 1.41 | 28.28 | 9.90 | 10.61 |
| Splicing factor 3B subunit 2 | Q13435 | SF3B2 | 100 kDa | 0.18 | [] | 6 | 7 | 43 | 33 | 14 | 61 | 24 | 11 | 27 | 29 | 0.71 | 7.07 | 33.23 | 9.19 | 1.41 |
| ATP-dependent RNA helicase A | Q08211 | DHX9 | 141 kDa | 0.35 | [] | 27 | 22 | 29 | 24 | 13 | 61 | 12 | 10 | 26 | 23 | 3.54 | 3.54 | 33.94 | 1.41 | 2.12 |
| Cullin-associated NEDD8-dissociated protein 1 | Q86VP6 | CAND1 | 136 kDa | 0.46 | [] | 26 | 21 | 41 | 36 | 13 | 74 | 12 | 17 | 32 | 32 | 3.54 | 3.54 | 43.13 | 3.54 | 0.00 |
| Protein PRRC2C | Q9Y520 (+2) | PRRC2C | 317 kDa | 0.049 | [] | 13 | 7 | 18 | 16 | 23 | 76 | 22 | 14 | 15 | 9 | 4.24 | 1.41 | 37.48 | 5.66 | 4.24 |
| Isoform 2 of Regulator of nonsense transcripts 1 | Q92900-2 | UPF1 | 123 kDa | 0.31 | [] | 15 | 14 | 26 | 24 | 12 | 71 | 7 | 12 | 9 | 14 | 0.71 | 1.41 | 41.72 | 3.54 | 3.54 |
| Catenin beta-1 | B4DGU4 (+1) | CTNNB1 | 85 kDa | < 0.00010 | AGS high, H446 low, HCT116 high, PC3 low, SHSY5S low | 33 | 25 | 23 | 21 | 27 | 50 | 4 | 8 | 10 | 7 | 5.66 | 1.41 | 16.26 | 2.83 | 2.12 |
| Heterogeneous nuclear ribonucleoprotein U | Q00839 | HNRNPU | 91 kDa | 0.099 | [] | 15 | 9 | 19 | 16 | 14 | 44 | 13 | 10 | 28 | 24 | 4.24 | 2.12 | 21.21 | 2.12 | 2.83 |
| Interleukin enhancer-binding factor 3 | Q12906 | ILF3 | 95 kDa | 0.003 | AGS low, H446 high, HCT116 low, PC3 low, SHSY5S low | 15 | 10 | 32 | 28 | 14 | 33 | 16 | 17 | 17 | 17 | 3.54 | 2.83 | 13.44 | 0.71 | 0.00 |
| Replication protein A 70 kDa DNA-binding subunit | P27694 | RPA1 | 68 kDa | 0.0024 | AGS low, H446 high, HCT116 low, PC3 low, SHSY5S high | 7 | 5 | 34 | 32 | 12 | 24 | 8 | 17 | 35 | 26 | 1.41 | 1.41 | 8.49 | 6.36 | 6.36 |
| Matrin-3 | A0A0R4J2E8 (+1) | MATR3 | 95 kDa | 0.53 | [] | 11 | 10 | 17 | 17 | 6 | 31 | 10 | 12 | 21 | 19 | 0.71 | 0.00 | 17.68 | 1.41 | 1.41 |
| Ras GTPase-activating protein-binding protein 1 | Q13283 | G3BP1 | 52 kDa | 0.0013 | AGS low, H446 high, HCT116 high, PC3 low, SHSY5S low | 8 | 9 | 31 | 28 | 17 | 46 | 8 | 8 | 13 | 10 | 0.71 | 2.12 | 20.51 | 0.00 | 2.12 |
| 40S ribosomal protein S4, X isoform | P62701 | RPS4X | 30 kDa | 0.26 | [] | 8 | 11 | 16 | 15 | 18 | 22 | 5 | 9 | 13 | 18 | 2.12 | 0.71 | 2.83 | 2.83 | 3.54 |
| Nucleolin | P19338 | NCL | 77 kDa | 0.26 | [] | 9 | 8 | 17 | 20 | 17 | 17 | 11 | 4 | 25 | 20 | 0.71 | 2.12 | 0.00 | 4.95 | 3.54 |
| Casein kinase II subunit alpha | E7EU96 | CSNK2A1 | 45 kDa | 0.18 | [] | 12 | 16 | 27 | 25 | 19 | 29 | 4 | 16 | 12 | 14 | 2.83 | 1.41 | 7.07 | 8.49 | 1.41 |
| Isoform 2 of Putative ATP-dependent RNA helicase DHX30 | Q7L2E3-2 | DHX30 | 136 kDa | 0.32 | [] | 11 | 6 | 10 | 11 | 9 | 50 | 6 | 6 | 11 | 15 | 3.54 | 0.71 | 28.99 | 0.00 | 2.83 |
| Pre-mRNA-splicing factor ATP-dependent RNA helicase DHX15 | O43143 | DHX15 | 91 kDa | 0.035 | [] | 8 | 6 | 26 | 21 | 9 | 19 | 10 | 2 | 22 | 23 | 1.41 | 3.54 | 7.07 | 5.66 | 0.71 |
| DNA repair protein XRCC1 | P18887 | XRCC1 | 69 kDa | 0.004 | AGS low, H446 low, HCT116 low, PC3 low, SHSY5S high | 11 | 11 | 14 | 13 | 6 | 12 | 8 | 13 | 51 | 33 | 0.00 | 0.71 | 4.24 | 3.54 | 12.73 |
| Zinc finger protein 609 | O15014 | ZNF609 | 151 kDa | 0.057 | [] | 5 | 10 | 13 | 20 | 4 | 28 | 7 | 5 | 33 | 28 | 3.54 | 4.95 | 16.97 | 1.41 | 3.54 |
| Poly(U)-binding-splicing factor PUF60 (Fragment) | A0A0J9YVP6 (+4) | PUF60 | 57 kDa | 0.33 | [] | 10 | 11 | 19 | 21 | 16 | 15 | 11 | 13 | 8 | 11 | 0.71 | 1.41 | 0.71 | 1.41 | 2.12 |
| Heterogeneous nuclear ribonucleoproteins C1/C2 | B2R5W2 (+2) | HNRNPC | 32 kDa | 0.65 | [] | 8 | 6 | 10 | 8 | 7 | 14 | 9 | 10 | 19 | 7 | 1.41 | 1.41 | 4.95 | 0.71 | 8.49 |
| Spliceosome RNA helicase DDX39B | Q13838 (+1) | DDX39B | 49 kDa | 0.012 | [] | 6 | 7 | 22 | 29 | 4 | 9 | 4 | 9 | 18 | 22 | 0.71 | 4.95 | 3.54 | 3.54 | 2.83 |
| ATP-dependent RNA helicase DDX39A | O00148 | DDX39A | 49 kDa | 0.019 | [] | 3 | 7 | 21 | 22 | 4 | 8 | 4 | 9 | 13 | 18 | 2.83 | 0.71 | 2.83 | 3.54 | 3.54 |
| Thyroid hormone receptor-associated protein 3 | Q9Y2W1 | THRAP3 | 109 kDa | 0.59 | [] | 7 | 6 | 11 | 12 | 21 | 13 | 15 | 10 | 10 | 13 | 0.71 | 0.71 | 5.66 | 3.54 | 2.12 |
| Isoform 2 of ELAV-like protein 1 | Q15717-2 | ELAVL1 | 39 kDa | 0.021 | [] | 8 | 8 | 12 | 7 | 8 | 21 | 12 | 13 | 7 | 6 | 0.00 | 3.54 | 9.19 | 0.71 | 0.71 |
| Heterogeneous nuclear ribonucleoprotein A3 | P51991 | HNRNPA3 | 40 kDa | 0.2 | [] | 7 | 10 | 15 | 14 | 5 | 13 | 15 | 12 | 8 | 13 | 2.12 | 0.71 | 5.66 | 2.12 | 3.54 |
| Heterogeneous nuclear ribonucleoprotein L | P14866 | HNRNPL | 64 kDa | 0.37 | [] | 10 | 7 | 19 | 14 | 3 | 11 | 14 | 6 | 15 | 9 | 2.12 | 3.54 | 5.66 | 5.66 | 4.24 |
| 60S ribosomal protein L7 | P18124 | RPL7 | 29 kDa | 0.99 | [] | 4 | 11 | 8 | 11 | 7 | 13 | 5 | 8 | 10 | 10 | 4.95 | 2.12 | 4.24 | 2.12 | 0.00 |
| 40S ribosomal protein S2 | P15880 | RPS2 | 31 kDa | 0.13 | [] | 8 | 3 | 13 | 7 | 11 | 21 | 6 | 3 | 14 | 9 | 3.54 | 4.24 | 7.07 | 2.12 | 3.54 |
| Serine/arginine-rich-splicing factor 1 | J3KTL2 (+1) | SRSF1 | 28 kDa | 0.0099 | [] | 8 | 9 | 10 | 11 | 5 | 11 | 8 | 10 | 16 | 16 | 0.71 | 0.71 | 4.24 | 1.41 | 0.00 |
| Polymerase delta-interacting protein 3 | Q9BY77 | POLDIP3 | 46 kDa | 0.077 | [] | 10 | 11 | 13 | 19 | 4 | 7 | 8 | 12 | 17 | 12 | 0.71 | 4.24 | 2.12 | 2.83 | 3.54 |
| 40S ribosomal protein S17 | P08708 | RPS17 | 16 kDa | 0.43 | [] | 6 | 5 | 11 | 5 | 9 | 16 | 5 | 5 | 20 | 7 | 0.71 | 4.24 | 4.95 | 0.00 | 9.19 |
| Double-stranded RNA-binding protein Staufen homolog 1 | O95793 (+1) | STAU1 | 63 kDa | 0.21 | [] | 6 | 4 | 14 | 9 | 4 | 16 | 6 | 10 | 5 | 6 | 1.41 | 3.54 | 8.49 | 2.83 | 0.71 |
| WD repeat-containing protein 5 | P61964 | WDR5 | 37 kDa | 0.36 | [] | 3 | 8 | 15 | 14 | 2 | 9 | 8 | 4 | 6 | 14 | 3.54 | 0.71 | 4.95 | 2.83 | 5.66 |
| Peroxisomal multifunctional enzyme type 2 | P51659 | HSD17B4 | 80 kDa | 0.64 | [] | 8 | 11 | 7 | 11 | 3 | 7 | 3 | 15 | 8 | 9 | 2.12 | 2.83 | 2.83 | 8.49 | 0.71 |
| 40S ribosomal protein S3a | P61247 | RPS3A | 30 kDa | 0.015 | [] | 8 | 8 | 17 | 21 | 15 | 30 | 5 | 10 | 29 | 20 | 0.00 | 2.83 | 10.61 | 3.54 | 6.36 |
