# Supplementary material for: Characterization of the interactome profiling of Mycoplasma fermentans DnaK in cancer cells reveals interference with key cellular pathways
Source: Front Microbiol. 2022 Oct 28;13:1022704. doi: 10.3389/fmicb.2022.1022704 (PMC9651203; doi:10.3389/fmicb.2022.1022704)
Supplement: Supplementary file 1 [file Data_Sheet_1.ZIP › Supplemental/Table S3.pdf]

Table S3: List of the top 10 Molecular Complex Detection (MCODE) components obtained from the protein-protein interaction analysis. The top 3 GO terms for each MCODE are listed.

MCODE	GO	Description	p-value
MCODE 1	R-HSA-72163	mRNA splicing- Major Pathway	-88.8
	R-HSA-72172	mRNA splicing	-87.9
	R-HSA-72203	Processing of Capped Intron-Containing pre-mRNA	-83.1
MCODE 2	R-HSA-975956	Nonsense Mediated Decay (NMD) independent from the exon junction complex	-68.3
	R-HSA-156827	L13a-mediated translational silencing of Ceruloplasmin expression	-66
	R-HSA-975957	Nonsense Mediated Decaly (NMD) enhanced by the Exon Junction Complex	-65.5
MCODE 3	WP4320	the effect of Progerin on the involved genes in Hutchinson-Gilford Progeria Syndrome	-11.1
	R-HSA-6807070	PTEN regulation	-10.2
	CORUM:282	SNF2h-cohesin-NuRD complex	-10
MCODE 4	CORUM:302	INO80 chromatin remodeling complex	-10.4
	M42	PID DNAPK pathway	-10
	CORUM:2720	Casein Kinase II (beta-dimer, alpha, alpha')	-9.9
MCODE 5	CORUM:1332	Large Dhrosha Complex	-7.2
	M258	PID BARD1 Pathway	-6.7
	GO:0000398	mRNA splicing, via spliceosome	-4.8
MCODE 6	GO:0006338	chromatin remodeling	-6.3
	GO:0061013	regulation of mRNA catabolic process	-6
	GO:0032870	cellular response to hormone stimulus	-5.5
MCODE 7	GO:0000398	mRNA splicing, via spliceosome	-7
	GO:0000377	RNA splicing, via transesterification reactions with bulged adenosine as nucluophile	-7
	GO:0000375	RNA splicing, via transesterification reactions	-6.9
MCODE 8	R-HSA-73933	Resolution of abasic sites (AP sites)	-15
	WP4946	DNA repair pathways full network	-14.6
	R-HSA-5693538	Homology Direct Repair	-14.2
MCODE 9	GO:0034063	stress granule assembly	-7.6
	GO:0017148	negative regulation of translation	-4.7
	GO:0034249	negative regulation of cellular amide metabolic process	-4.5
MCODE 10	GO:0071824	protein-DNA complex subunit organization	-11.2
	GO:0034728	nucleosome organization	-9.6
	GO:0006333	chromatin assembly or disassembly	-9.5

p-value p-value in Log base 10
