# Supplementary figures and images for: Characterization of the interactome profiling of Mycoplasma fermentans DnaK in cancer cells reveals interference with key cellular pathways
Source: Front Microbiol. 2022 Oct 28;13:1022704. doi: 10.3389/fmicb.2022.1022704 (PMC9651203; doi:10.3389/fmicb.2022.1022704)

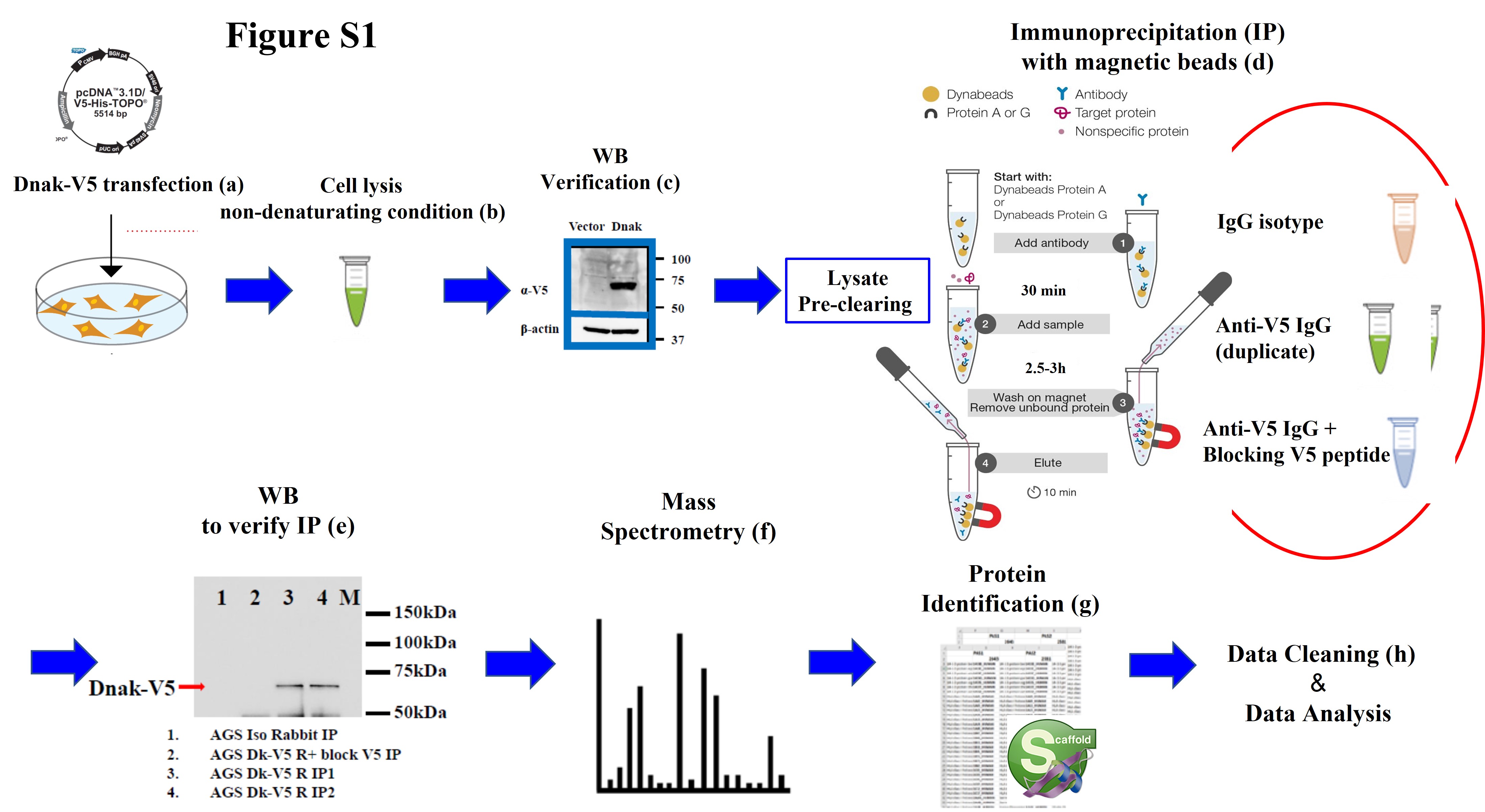

Supplement: Supplementary file 1 [file Data_Sheet_1.ZIP › Supplemental/FigS1.jpg]

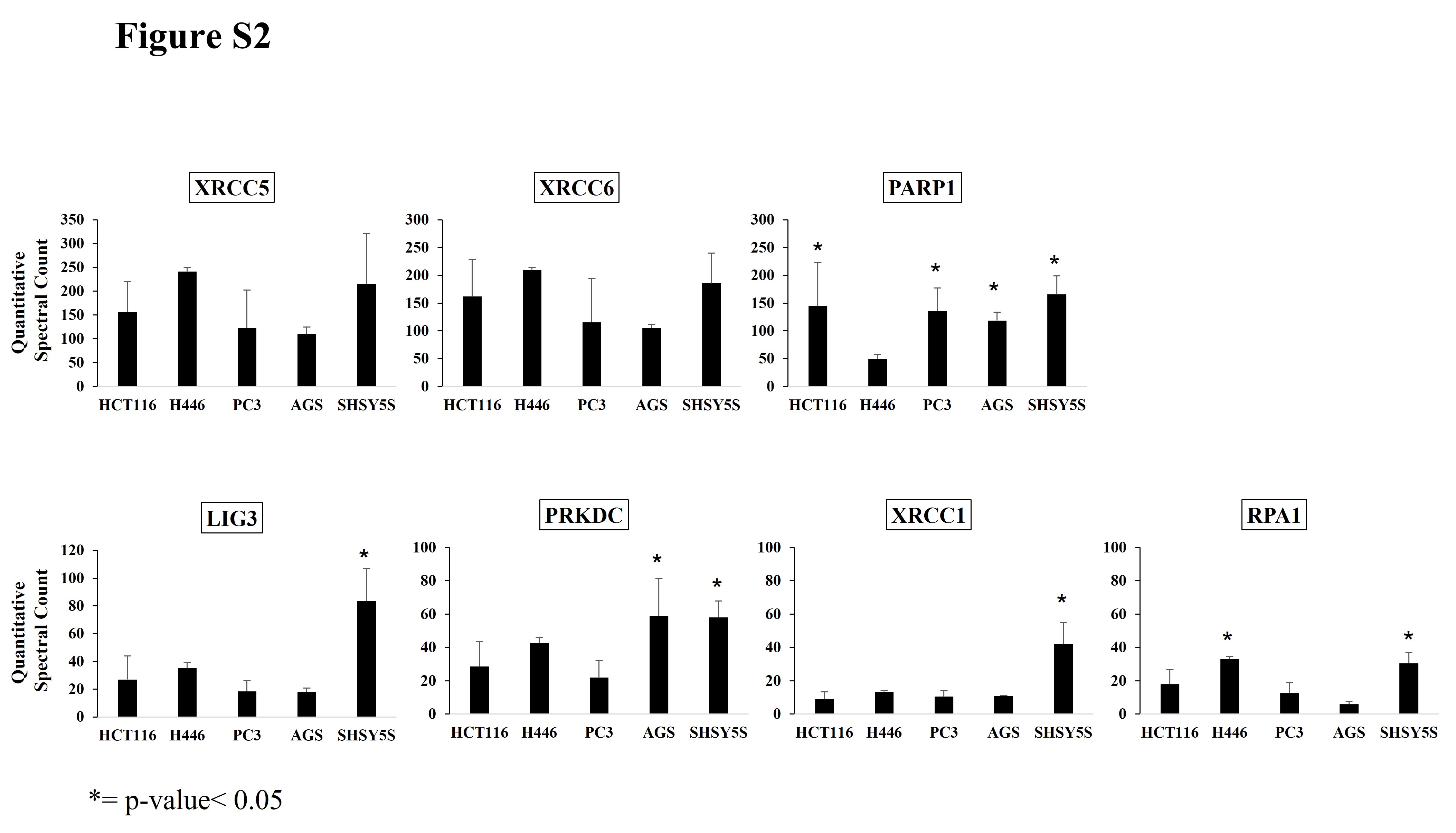

Supplement: Supplementary file 1 [file Data_Sheet_1.ZIP › Supplemental/FigS2.jpg]

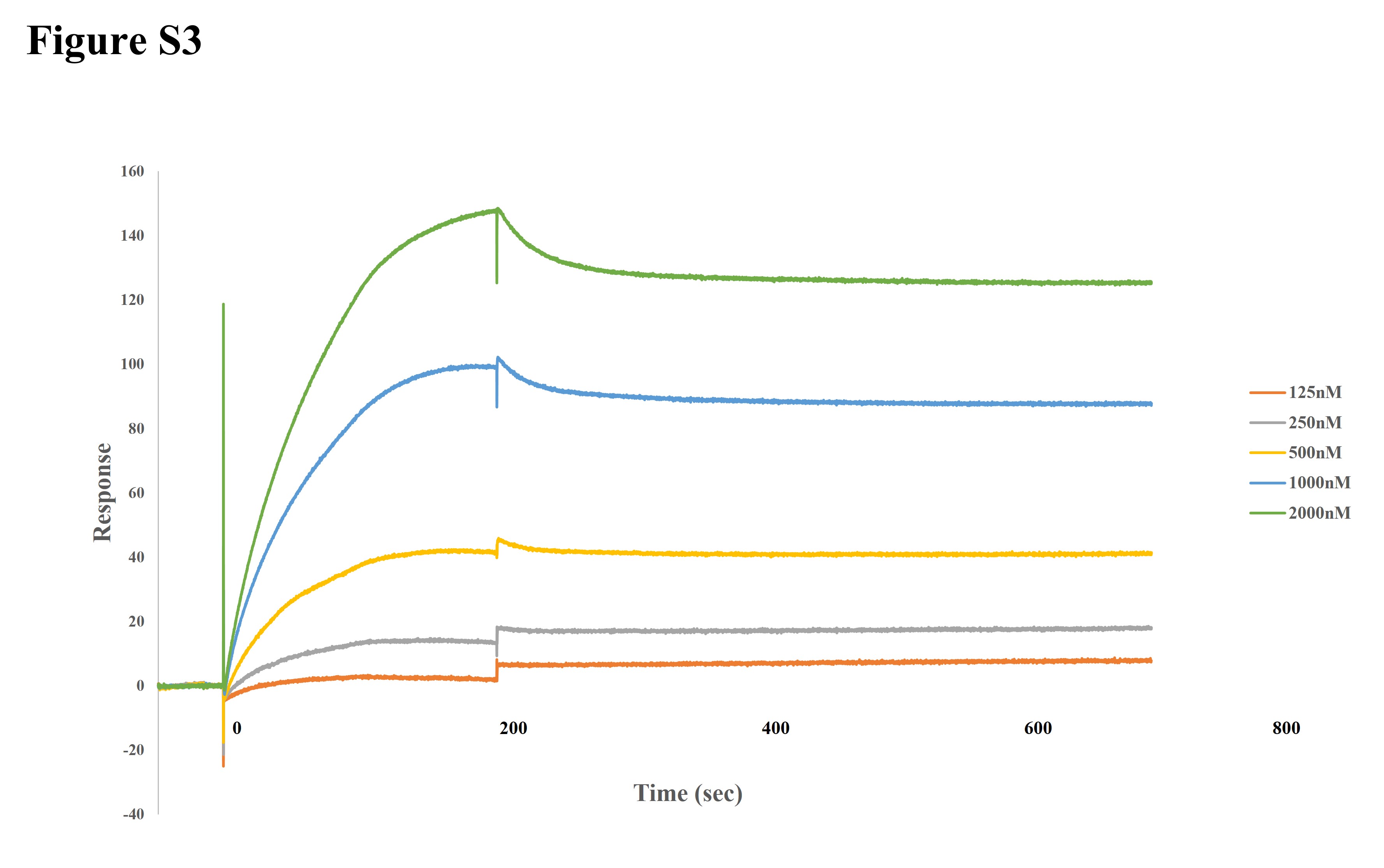

Supplement: Supplementary file 1 [file Data_Sheet_1.ZIP › Supplemental/FigS3.jpg]
